# The Gut Microbial Diversity of Newly Diagnosed Diabetics but Not of Prediabetics Is Significantly Different from That of Healthy Nondiabetics

**DOI:** 10.1128/mSystems.00578-19

**Published:** 2020-03-31

**Authors:** Akshay H. Gaike, Dhiraj Paul, Shrikant Bhute, Dhiraj P. Dhotre, Pranav Pande, Smitha Upadhyaya, Yugandhar Reddy, Ramya Sampath, Debjani Ghosh, D. Chandraprabha, Jhankar Acharya, Gautam Banerjee, Vilas P. Sinkar, Saroj S. Ghaskadbi, Yogesh S. Shouche

**Affiliations:** a National Centre for Microbial Resource, National Centre for Cell Science, Pune, India; b Unilever R&D, Whitefield, Bangalore, India; c Department of Zoology, Savitribai Phule Pune University, Pune, India; University of California, Irvine

**Keywords:** 16S rRNA gene, T2D, driver genera, gut microbiome, newly diagnosed diabetics, prediabetes, serum biomarkers, total antioxidants

## Abstract

Type 2 diabetes (T2D) is a complex metabolic syndrome characterized by insulin dysfunction and abnormalities in glucose and lipid metabolism. The gut microbiome has been recently identified as an important factor for development of T2D. In this study, a total of 102 subjects were recruited, and we have looked at the gut microbiota of prediabetics (PreDMs) (*n* = 17), newly diagnosed diabetics (NewDMs) (*n* = 11), and diabetics on antidiabetic treatment (KnownDMs) (*n* = 39) and compared them with healthy nondiabetics (ND) (*n* = 35). Twenty-five different serum biomarkers were measured to assess the status of diabetes and their association with gut microbiota. Our analysis revealed nine different genera as differentially abundant in four study groups. Among them, *Akkermansia, Blautia,* and *Ruminococcus* were found to be significantly (*P* < 0.05) decreased, while *Lactobacillus* was increased in NewDMs compared to ND and recovered in KnownDMs. *Akkermansia* was inversely correlated with HbA1c and positively correlated with total antioxidants. Compared to ND, there was increased abundance of *Megasphaera*, *Escherichia*, and *Acidaminococcus* and decreased abundance of *Sutterella* in KnownDMs. Among many taxa known to act as community drivers during disease progression, we observed genus *Sutterella* as a common driver taxon among all diabetic groups. On the basis of the results of random forest analysis, we found that the genera *Akkermansia* and *Sutterella* and that the serum metabolites fasting glucose, HbA1c, methionine, and total antioxidants were highly discriminative factors among studied groups. Taken together, our data revealed that gut microbial diversity of NewDMs but not of PreDMs is significantly different from that of ND. Interestingly, after antidiabetic treatment, the microbial diversity of KnownDMs tends to recover toward that of ND.

**IMPORTANCE** Gut microbiota is considered to play a role in disease progression, and previous studies have reported an association of microbiome dysbiosis with T2D. In this study, we have attempted to investigate gut microbiota of ND, PreDMs, NewDMs, and KnownDMs. We found that the genera *Akkermansia* and *Blautia* decreased significantly (*P* < 0.05) in treatment-naive diabetics and were restored in KnownDMs on antidiabetic treatment. To the best of our knowledge, comparative studies on shifts in the microbial community in individuals of different diabetic states are lacking. Understanding the transition of microbiota and its association with serum biomarkers in diabetics with different disease states may pave the way for new therapeutic approaches for T2D.

## Abstract (precis)

Gut microbiota is considered to play a role in disease progression, and previous studies have reported an association of microbiome dysbiosis with T2D. In this study, we have attempted to investigate gut microbiota of ND, PreDMs, NewDMs, and KnownDMs. We found that the genera *Akkermansia* and *Blautia* decreased significantly (*P* < 0.05) in treatment-naive diabetics and were restored in KnownDMs on antidiabetic treatment. To the best of our knowledge, comparative studies on shifts in the microbial community in individuals of different diabetic states are lacking. Understanding the transition of microbiota and its association with serum biomarkers in diabetics with different disease states may pave the way for new therapeutic approaches for T2D.

## INTRODUCTION

Type 2 diabetes (T2D) is a global epidemic; it has been estimated that 450 million people will be affected by this metabolic disorder by 2025 (1). In addition to host genetics, environmental factors, and sedentary lifestyle (2), gut microbiota has turned out to be an important contributor for development of T2D (3–6). T2D is characterized by hyperglycemia, insulin resistance, and insufficient insulin secretion and is associated with disturbed glucose, lipid, and amino acid metabolism (7–9). In particular, high levels of branched-chain amino acids (BCAA) and aromatic amino acids (AAA) have been associated with a high risk of developing insulin resistance (10). Oxidative stress is also known to be involved in the development of insulin resistance and, more importantly, in the development of diabetic complications (11). Over the past decade, significant efforts are being made to map the structural and functional attributes of human gut microbial communities to understand the disease progression (12, 13). Throughout life, the gut microbiota acts as a sensory hub, responding to both intrinsic and extrinsic stimuli affecting host physiology within and outside the gut (14). Disruption of a delicate balance among the gut microbes has been linked to the development of metabolic diseases and particularly T2D (5), obesity (15), and cardiovascular disorders (16). Most of the earlier studies have reported differences between the gut microbiome of diabetics, prediabetics, and healthy nondiabetic individuals (6, 17), and very few have examined gut microbiome of treatment-naive T2D individuals (18, 19).

In this study, we have analyzed the gut microbiome of ND, PreDMs, NewDMs, and KnownDMs to understand and identify differences in the microbial community associated with T2D and prediabetes. In addition, we also looked at the community changes in microbial association networks and identified driver genera for the transition from healthy (control) to diabetic state. Further, we analyzed the association of a wide array of serum biomarkers with genera, which were differentially abundant and were also found to be driver taxa.

## RESULTS

### Analysis of serum biomarkers.

To understand pathophysiological condition of diabetic subjects, 25 different serum biomarkers relevant to T2D were assessed in all groups. Compared to ND, fasting glucose level in PreDMs (*P = *0.0006), NewDMs (*P* < 0.0001), and KnownDMs (*P* < 0.0001) and HbA1c level in PreDMs (*P < *0.0001), NewDMs (*P < *0.0001), and KnownDMs (*P < *0.0001) were found to be significantly higher. Similarly, in the lipid profile, triglycerides and very low-density lipoprotein (VLDL) cholesterol increased significantly in NewDMs (*P* = 0.0065 and *P = *0.0056, respectively) and KnownDMs (*P = *0.039 and *P = *0.035, respectively), but not in PreDMs compared to ND. High-density lipoprotein (HDL) cholesterol decreased significantly in NewDMs (*P = *0.029) and KnownDMs (*P = *0.022) compared to ND. The level of apolipoprotein A1 (*P = *0.0003) was found to be significantly lower in NewDMs compared to ND, while it remained unchanged in PreDMs and KnownDMs (Table 1). The level of folic acid was found to be increased significantly only in KnownDMs compared to ND (*P = *0.031). Eight different amino acids were analyzed in the serum of all four groups. Among these eight amino acids, tyrosine (*P = *0.0001), tryptophan (*P* < 0.0001), valine (*P = *0.0009), leucine (*P* < 0.0001), and methionine (*P = *0.014) were found to be significantly increased, while histidine (*P = *0.02) was found to be decreased in NewDMs compared to ND. In PreDMs, only methionine was found to be decreased (*P = *0.033) compared to ND. In KnownDMs, only four amino acids, namely, tyrosine (*P* < 0.0001), tryptophan (*P = *0.0036), isoleucine (*P = *0.003) and leucine (*P = *0.014) were found to be significantly increased compared to ND. Interleukin 6 (IL-6), a marker of inflammation was significantly higher in all three groups, namely, PreDMs (*P = *0.0098), NewDMs (*P = *0.0014), and KnownDMs (*P* < 0.0001) compared to ND, and lipopolysaccharide (LPS), a bacterial endotoxin, was found to be significantly increased only in NewDMs (*P = *0.0041) compared to ND. Adiponectin did not change in any group compared to ND. Lipid peroxides, a marker for oxidative damage, were found to be significantly increased in both NewDMs (*P = *0.0008) and KnownDMs (*P = *0.0014) but not in PreDMs compared to ND, while total antioxidant capacity was found to be significantly (*P = *0.029) low only in NewDMs compared to ND (Table 1).

**TABLE 1:** Serum biomarkers and anthropometric parameters of the participants^*a*^

Characteristic^*b*^	ND (*n* = 35) (17 M, 18 F)			PreDMs (*n* = 17) (6 M, 11 F)			NewDMs (*n* = 11) (9 M, 2 F)			KnownDMs (*n* = 39) (28 M, 11 F)		
	Mean ± SD	Median	Range	Mean ± SD	Median	Range	Mean ± SD	Median	Range	Mean ± SD	Median	Range
Age (yr)	37 ± 7.6	34	30 − 59	46 ± 9.6*	49	32 − 59	44.81 ± 7.46*	44	34 − 60	52 ± 6.5*	52	33 − 60
Wt (kg)	67.58 ± 10.13	67.3	51 − 92	69.96 ± 11.62	69	55 − 94.34	79.10 ± 15.74*	78	48 − 98.6	70.2 ± 10.45	68	51.4 − 104.8
BMI (kg/m^2^)	24.73 ± 2.65	24.77	18.84 − 29.97	26.35 ± 4.73	26.67	17.94 − 33.77	28.34 ± 3.48*	29.39	20.66 − 32.04	26.1 ± 3.23	25.60	20.33 − 37.94
Fasting glucose (mg/dl)	91.65 ± 8.07	91	75 − 115	101.06 ± 9.91*	100	83 − 142	163.18 ± 57.26*	144	95 − 275	154.07 ± 43.55*	145	89 − 272
HbA1c (%)	5.37 ± 0.21	5.4	4.9 − 5.7	5.98 ± 0.18*	6.0	5.8 − 6.4	8.5 ± 2.29*	7.4	6.6 − 12.7	8.058 ± 1.19*	7.9	6.5 − 10.9
Total cholesterol (mg/dl)	170.4 ± 41.2	161	115 − 263	171 ± 23.36	176	129 − 214	182.54 ± 39.18	184	124 − 251	159.87 ± 39.004	157	76 − 247
Triglycerides (mg/dl)	109.65 ± 70.76	82	39 − 389	93.8 ± 32.59	103	36 − 143	166.54 ± 65.86*	168	65 − 581	144.10 ± 87.46*	116	50 − 395
HDL cholesterol (mg/dl)	46 ± 10.28	45	22 − 75	44.26 ± 6.81	44	37 − 56	39.6± 8.3*	40	28 − 62	40.89 ± 9.46*	39	25 − 70
VLDL cholesterol (mg/dl)	21.91 ± 14.10	16	8 − 78	18.66 ± 6.56	21	7 − 29	33.36 ± 13.1*	34	13 − 56	28.82 ± 17.46*	23	10 − 79
LDL cholesterol (mg/dl)	102.48 ± 33.27	98	16 − 172	108.066 ± 20.74	117	68 − 150	109.54 ± 33.6	110	47 − 162	90.15 ± 34.48	92	25 − 182
Apolipoprotein A1 (mg/dl)	134.82 ± 18.81	135	99 − 175	132.13 ± 14.38	128	99 − 154	111.90 ± 16.44*	110	91 − 151	130.71 ± 21.20	126	96 − 199
Apolipoprotein B (mg/dl)	87.54 ± 20.34	83	58 − 134	89.6 ± 14.27	92	62 − 121	101.81 ± 24.22	102	60 − 137	82.33 ± 22.36	81	36 − 138
Vitamin B_12_ (pg/ml)	251.6 ± 193.77	195	83 − 1048	290.26 ± 197.60	179	111 − 801	209.54 ± 73.47	189	131 − 386	356.17 ± 415.78	202	83 − 1,706
Folic acid (ng/ml)	6.61 ± 3.6	6	1.4 − 15.6	7.6 ± 3.073	6.5	3.4 − 13.9	8.18 ± 4.99	6.8	2.2 − 20	9.00 ±4.85*	8.1	1.8 − 20
Homocysteine (μmol/liter)	19.4 ± 11.6	15.3	5.26 − 50	18.13 ± 13.41	14.7	7.85 − 50	22.35 ± 13.42	15.41	9.49 − 50	19.42 ±11.35	16.91	6.49 − 50.1
Histidine (μg/0.1 ml)	1.75 ± 0.79	1.67	0.67 − 3.28	1.58 ± 0.56	1.46	0.8 − 2.36	1.08 ± 0.34*	0.91	0.69 − 1.60	1.81 ± 0.72	1.76	0.46 − 3.38
Tyrosine (μg/0.1 ml)	4.52 ± 1.44	4.46	1.18 − 9.1	3.94 ± 1.018	3.84	2.19 − 6.06	6.5 ± 1.23*	6.3	4.21 − 8.20	5.74 ± 1.27*	5.91	3.4 − 8.53
Tryptophan (μg/0.1ml)	0.85 ± 0.34	0.8	0.43 − 1.97	0.90 ± 0.30	0.93	0.49 − 2.02	1.65 ± 0.38*	1.66	1.18 − 2.30	1.069 ± 0.37*	1.02	0.53 − 2.1
Methionine (μg/0.1 ml)	0.33 ± 0.10	0.35	0.14 − 0.59	0.28 ± 0.058*	0.29	0.17 − 0.77	0.46 ± 0.13*	0.45	0.31 − 0.69	0.33 ± 0.094	0.33	0.17 − 0.63
Valine (μg/0.1 ml)	3.039 ± 0.67	3.095	0.88 − 4.1	2.73 ± 0.64	2.84	1.8 − 4.21	4.01 ± 0.78*	3.99	2.75 − 5.10	3.231 ± 0.58	3.19	2.15 − 4.29
Phenylalanine (μg/0.1 ml)	2.8 ± 1.10	2.56	1.11 − 5.2	2.43 ± 0.81	2.42	0.96 − 3.85	3.38 ± 0.41	3.35	2.88 − 4.0	2.86 ± 0.74	2.72	1.58 − 4.66
Isoleucine (μg/0.1 ml)	1.18 ± 0.284	1.15	0.64 − 1.76	1.052 ± 0.25	1.02	0.63 − 1.43	1.21 ± 0.24	1.19	0.86 − 1.60	1.38 ± 0.26*	1.37	0.79 − 2.0
Leucine (μg/0.1 ml)	1.80 ± 0.51	1.78	0.68 − 3.05	1.6 ± 0.44	1.79	0.92 − 2.51	2.755 ± 0.48*	2.67	1.94 − 3.40	2.17 ± 0.56*	2.06	1.28 − 3.46
Serum IL-6 (pg/ml)	1.18 ± 0.92	0.95	0.36 − 5.27	1.68 ± 0.73*	1.43	0.2 − 3.12	2.47 ± 1.69*	2.39	0.51 − 6.67	2.990 ± 3.25*	2.0	0.82 − 15.21
Serum adiponectin (μg/ml)	5.6 ± 3.31	5.00	0.75 − 12.79	6.47 ± 2.71	5.08	2.23 − 10.33	3.75 ± 0.94	3.76	2.37 − 5.03	5.40 ± 2.71	1.95	1.72 − 12.04
Serum LPS (ng/ml)	102.63 ± 129.11	48.15	5.31 − 531.8	143.89 ± 162.75	70.89	23.02 − 507.25	222.98 ± 124.54*	221.98	0.45 − 484.72	82.88 ± 88.90	52.24	10.03 − 464.11
Total antioxidant (μM Trolox equivalent)	0.24 ± 0.10	0.21	0.07 − 0.4	0.24 ± 0.087	0.23	0.16 − 0.33	0.17 ± 0.05*	0.21	0.08 − 0.3	0.24 ± 0.06	0.26	0.07 − 0.51
Lipid peroxides (nmol of TBARs/ml)	2.15 ± 1.40	2.57	1.12 − 19.28	1.85 ± 0.57	2.56	1.09 − 5.57	10.44 ± 6.84*	1.71	1.06 − 10.89	2.81 ± 1.74*	1.88	0.55 − 8.33

a The number of participants (*n*) and number of males (M) and females (F) are shown for the four groups of participants. Values that are significantly different (*P* < 0.05) from the value for the control (ND) are indicated by an asterisk.

b BMI, body mass index. BMI categories and reference values are as follows: underweight, less than 18.5 kg/m^2^; normal, between 18.5 and 25 kg/m^2^; overweight, between 25 kg/m^2^ and 29.9 kg/m^2^; obese, 30 kg/m^2^ or higher. TBARs, thiobarbituric acid-reactive substances.

### Microbial diversity analysis and identification of differentially abundant microbial signatures.

**FIG 1:**
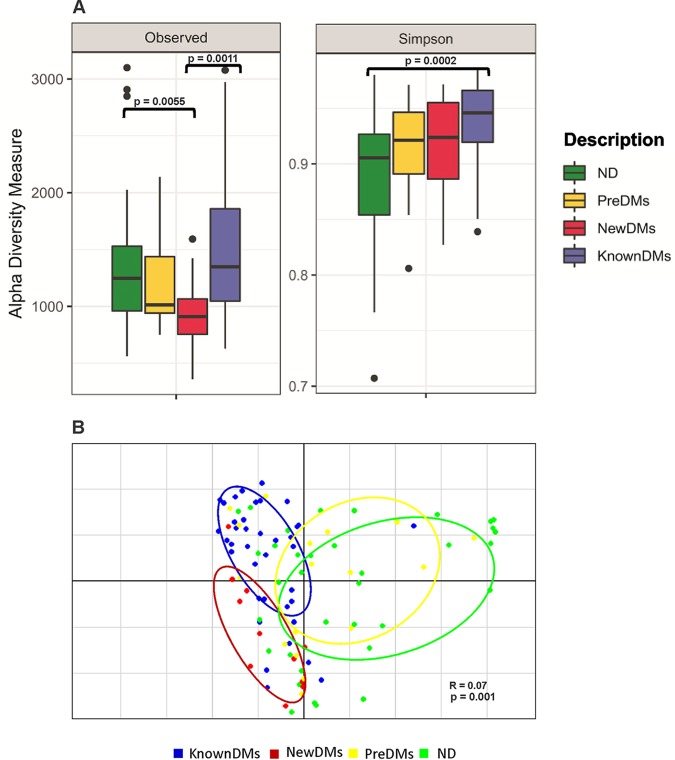
(A) Alpha diversity analysis across all four groups, ND, PreDMs, NewDMs, and KnownDMs. Alpha diversity measures, including the number of observed OTUs and Simpson indices, revealed statistically significant differences among diabetic groups compared to ND. Pairwise comparisons were analyzed using a Mann-Whitney nonparametric test. *P < *0.05. Solid black circles indicate the sample outliers. (B) Beta diversity analysis of the microbiota across four study groups. Principal-coordinate analysis (PCoA) based on generalized UniFrac distances between the samples, followed by PERMANOVA test (*R* = 0.07 and *P* = 0.001).

**FIG 2:**
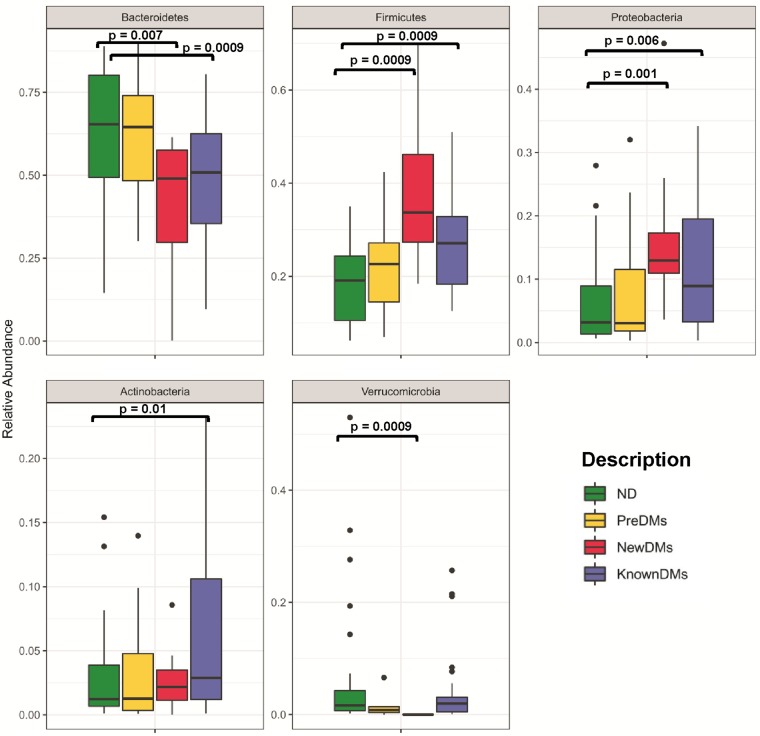
Comparison of differentially abundant significant phyla among all study groups. The mean difference test was performed for statistical significance with FDR correction by the DS-FDR method. *P* < 0.05.

**FIG 3:**
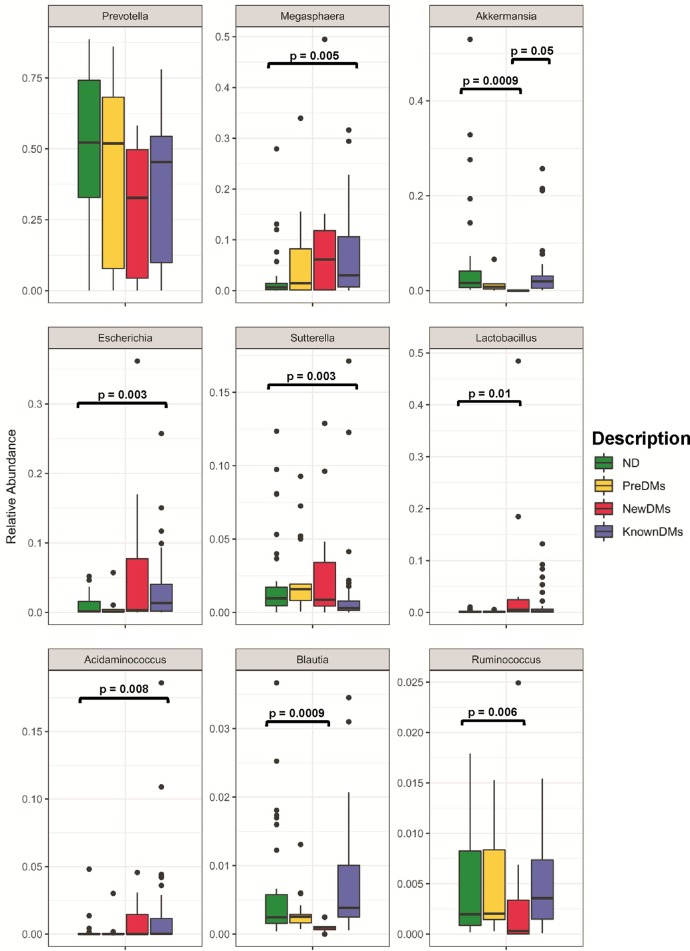
Comparison of differentially abundant significant genera among all study groups. The mean difference test was performed for statistical significance with FDR correction by the DS-FDR method. *P* < 0.05.

A total of nearly 44 million (43,902,890) high-quality sequences were retained after removal of low-quality sequences for taxonomic classification with average sequence reads of 430,420.49 ± 239,742.68 per sample (see Table S1 in the supplemental material). A total of 12,827 operational taxonomic units (OTUs) were observed from all four study groups after removing singleton OTUs. Taxonomic assignment was performed using a 97% similarity cutoff with Greengenes reference database v13_8. Good’s coverage of ≥99% indicated a high degree of sequence coverage. In alpha diversity analysis, nonparametric indices such as the number of observed OTUs for richness and Simpson index for evenness were calculated. The observed number of OTUs showed that alpha diversity decreased significantly in NewDMs compared to ND (*P = *0.0055) and KnownDMs (*P = *0.0011), whereas a significant difference was not observed between KnownDMs and ND (Fig. 1A). Simpson index showed a significant increase in alpha diversity only in KnownDMs compared to ND (*P = *0.0002) (Fig. 1A). Overall bacterial community composition was analyzed by using generalized UniFrac distances (20) followed by permutational multivariate analysis of variance (PERMANOVA) test (*R* = 0.07, *P = *0.001) (Fig. 1B). The distance matrix is combined with unweighted and weighted UniFrac distances in a common structure and therefore is able to provide a much wider range of biologically appropriate changes. Two distinct clusters of KnownDMs and NewDMs were observed, whereas PreDMs formed an overlapping cluster with ND, indicating that the bacterial diversity of PreDMs is similar to that of ND. Interestingly, the diversity cluster of KnownDMs was found to be close to ND compared to NewDMs. Significant differences in bacteria belonging to five phyla, namely, *Bacteroidetes*, *Firmicutes*, *Proteobacteria*, *Actinobacteria*, and *Verrucomicrobia*, were observed in the gut microbiota of diabetic subjects (Fig. 2). Bacteria belonging to the phyla *Firmicutes* and *Proteobacteria* were significantly increased, whereas those from *Bacteroidetes* were significantly reduced in NewDMs (*P = *0.0009 and log_2_ fold change [log_2_ FC] = 1.09, *P = *0.001 and log_2_ FC = 1.51, and *P = *0.007 and log_2_ FC = −0.62, respectively) and KnownDMs (*P = *0.0009 and log_2_ FC = 0.58, *P = *0.006 and log_2_ FC = 0.99, and *P = *0.0009 and log_2_ FC = −0.37, respectively) compared to ND (Fig. 2 and Table S2). The ratio of *Firmicutes* to *Bacteroidetes* was calculated for all study groups. It was 1:4.94 for ND and 1:4.24 for PreDMs, and it changed significantly in NewDMs to 1:1.49. In KnownDMs on antidiabetic treatment, it was found to be changed to 1:1.23 (Table S3). The phylum *Verrucomicrobia* was found to be significantly decreased in NewDMs compared to ND (*P = *0.0009 and log_2_ FC = −14.2). In KnownDMs, the phylum *Actinobacteria* was found to be significantly increased compared to ND (*P = *0.011 and log_2_ FC = 1.16). A total of 1,127 OTUs were found to be significantly different in four study groups (*P* < 0.05). Of these OTUs, 10 OTUs belong to genus *Akkermansia*, 36 to *Prevotella*, 74 to *Blautia*, 24 to *Ruminococcus*, 45 to *Escherichia*, 50 to *Lactobacillus*, 4 to *Megasphaera*, 3 to *Sutterella* and 5 to *Acidaminococcus* (Table S4). In all the study groups, 519 genera were identified after merging all the OTUs belonging to the same genus, though they differed in their abundance. Of these genera, *Prevotella*, *Megasphaera*, *Akkermansia*, *Escherichia*, *Sutterella*, *Lactobacillus*, *Acidaminococcus*, *Blautia*, and *Ruminococcus* were found to have higher abundance than other genera in all four groups (Fig. 3). In NewDMs, *Akkermansia*, *Blautia*, and *Ruminococcus* showed significantly decreased abundance (*P* = 0.0009 and log_2_ FC = −14.2, *P* = 0.0009 and log_2_ FC = −2.52, and *P* = 0.006 and log_2_ FC = −0.39, respectively), and a similar trend was observed for *Prevotella* (*P* = 0.054 and not significant), one of the dominant genera found in Indian gut (19, 21, 22), while *Lactobacillus* (*P* = 0.01 and log_2_ FC = 5.27) showed increased abundance compared to ND. Significantly increased abundance of *Megasphaera* (*P* = 0.005 and log_2_ FC = 1.42), *Escherichia* (*P* = 0.003 and log_2_ FC = 1.96), and *Acidaminococcus* (*P* = 0.008 and log_2_ FC = 2.90) and decreased abundance of *Sutterella* (*P* = 0.003 and log_2_ FC = −0.66), was observed in KnownDMs compared to ND. In KnownDMs, increased abundance of *Akkermansia* was observed compared to NewDMs (*P = *0.0009 and log_2_ FC = 13.48) (Fig. 3 and Table S2).

10.1128/mSystems.00578-19.1 TABLE S1 Sequence statistics for bioinformatic analysis. Download Table S1, PDF file, 0.2 MB. Copyright © 2020 Gaike et al. 2020 Gaike et al. This content is distributed under the terms of the Creative Commons Attribution 4.0 International license.

10.1128/mSystems.00578-19.2 TABLE S2 Comparison of differentially abundant genera and phyla using the discrete false-discovery rate method (ds-FDR). Download Table S2, XLSX file, 0.01 MB. Copyright © 2020 Gaike et al. 2020 Gaike et al. This content is distributed under the terms of the Creative Commons Attribution 4.0 International license.

10.1128/mSystems.00578-19.3 TABLE S3 Ratio of *Firmicutes* to *Bacteroidetes* in each study group. Download Table S3, XLSX file, 0.01 MB. Copyright © 2020 Gaike et al. 2020 Gaike et al. This content is distributed under the terms of the Creative Commons Attribution 4.0 International license.

10.1128/mSystems.00578-19.4 TABLE S4 Differentially abundant significant taxa across study groups. Comparison based on Kruskal Wallis test. *P* < 0.05. Download Table S4, XLSX file, 0.08 MB. Copyright © 2020 Gaike et al. 2020 Gaike et al. This content is distributed under the terms of the Creative Commons Attribution 4.0 International license.

### Random forest analysis.

We used random forest analysis to identify differentially abundant or most discriminant features of microbiome and serum metabolites associated with the disease. Analyzing the microbial features, we found that *Akkermansia* and *Sutterella* are highly discriminative genera among four study groups with the highest mean decrease score (see Fig. S1A in the supplemental material). Among the serum biomarkers, fasting glucose, HbA1c, methionine, and total antioxidants are found to be highly discriminative parameters with the highest mean decrease score among four study groups (Fig. S1B).

10.1128/mSystems.00578-19.8 FIG S1 Random forest analysis. (A) Top ten highly discriminative microbial genera. (B) Top ten highly discriminative serum biomarkers. The values on the *x* axis represent the mean decrease scores for discriminative factors. Download FIG S1, TIF file, 0.4 MB. Copyright © 2020 Gaike et al. 2020 Gaike et al. This content is distributed under the terms of the Creative Commons Attribution 4.0 International license.

### Taxonomic distributions of rare bacteria.

It is well-known that low-abundance “rare” members of the bacterial communities in any ecosystem, including the human gut, are extremely divergent and can play major roles in various metabolic processes (23). Therefore, we investigated “rare” phylotypes which have an abundance of less than 0.01% in the total population (23). In the community analysis, of the total number of OTUs identified for ND (8,216 OTUs), 7,723 OTUs of rare phylotypes included *Bacteroidetes* (64%), *Firmicutes* (17.6%), *Proteobacteria* (6%), and *Actinobacteria* (2.89%). In PreDMs, of the total number of OTUs identified (5,577 OTUs), 5,092 rare OTUs found included *Bacteroidetes* (61.9%), *Firmicutes* (22.2%), *Proteobacteria* (8.1%), and *Actinobacteria* (3.4%), whereas in NewDMs, of the total OTUs identified (3,679 OTUs), 3,220 rare OTUs were found containing *Bacteroidetes* (41.31%), *Firmicutes* (38.4%), *Proteobacteria* (16.14%), and *Actinobacteria* (2.6%). In KnownDMs, of the total OTUs identified (10,134 OTUs), 9,570 OTUs were found to be of rare phylotypes and included *Firmicutes* (55.1%), *Proteobacteria* (20%), *Bacteroidetes* (13.8%), and *Actinobacteria* (6.8%) (Table S5). On the basis of the results of this analysis, we observed that the number of rare OTUs increased in KnownDMs on antidiabetic treatment compared to all other groups.

10.1128/mSystems.00578-19.5 TABLE S5 Distribution of rare OTUs across four study groups. Download Table S5, PDF file, 0.2 MB. Copyright © 2020 Gaike et al. 2020 Gaike et al. This content is distributed under the terms of the Creative Commons Attribution 4.0 International license.

### Identification of driver genera between four study groups based on NetShift analysis.

**FIG 4:**
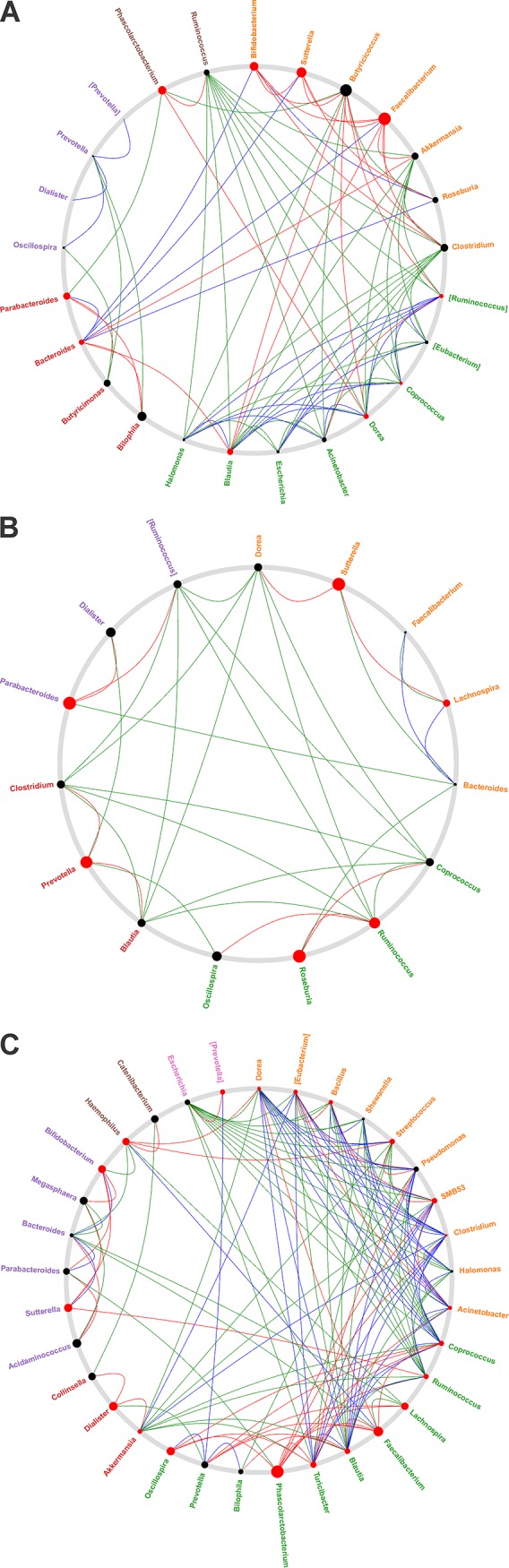
Identification of driver genera based on NetShift analysis. Comparison between ND (control) and PreDMs (case) (A), NewDMs (case) (B), and KnownDMs (case) (C). Driver genera are represented by red color nodes (circles) with a higher NESH score resulting in a bigger node. Edge (line) is assigned between the nodes; green represents microbial association only in the control, red represents association only in the case, and blue represents common microbial association of a node in the case and control.

We generated microbial association networks for ND, PreDMs, NewDMs, and KnownDMs followed by mining only statistically significant (*P* < 0.05) positive association networks separately using CCREPE (Compositionality Corrected by REnormalization and PErmutation) tool (http://huttenhower.sph.harvard.edu/ccrepe). To identify the driver genera between the case and control, NetShift workflow was performed (24). Driver genera can be identified based on the NESH score and node size. NESH is a Neighbor Shift score which represents directional changes in individual node associations, and a node represents each taxon. The node size is proportional to their respective NESH score, and a node is colored red if its betweenness increases from control to case. The nodes that are big and red are important community drivers (24). Comparison of ND (control) and PreDM (case) network (Fig. 4A) revealed *Bifidobacterium*, *Faecalibacterium*, *Sutterella*, and *Phascolarctobacterium* as the driver nodes (genera) with higher NESH scores (red color and bigger nodes), followed by *Bacteroides*, *Blautia*, *Dorea*, and *Parabacteroides* with low NESH scores (red color and smaller nodes) (Table S6). Among these driver genera, *Blautia* was found to be positively associated with major abundant genera such as *Akkermansia*, *Clostridium*, and *Ruminococcus*, along with other less abundant genera in ND (control). However, in PreDMs, *Blautia* showed positive association with *Bacteroides*, *Butyricicoccus*, and *Faecalibacterium* and not with *Akkermansia*, *Clostridium*, and *Ruminococcus* similar to ND, suggesting that *Blautia* may be a community driver for PreDMs. Another major driver *Sutterella*, which was found to be associated only with *Bacteroides* in ND, was associated with many other genera such as *Bacteroides*, *Bifidobacterium*, *Butyricicoccus*, *Faecalibacterium*, and *Roseburia* in PreDMs. Comparison of ND (control) with NewDMs (case) revealed that *Prevotella*, *Parabacteroides*, *Roseburia*, *Ruminococcus*, and *Sutterella* were found to have high NESH scored, indicating that these were the driver nodes (Fig. 4B and Table S6). *Sutterella*, one of the main drivers, was found to be associated with *Bacteroides* in ND and shifted its association in NewDMs with *Dorea* and *Lachnospira*. Another driver, *Prevotella*, showed association with *Dialister* and *Oscilospira* in ND, which was shifted to *Blautia* and *Clostridium* in NewDMs. Similarly, driver *Ruminococcus* was associated with *Blautia*, *Clostridium*, *Coprococcus*, and *Dorea* in the ND group and shifted its association with *Oscilospira* and *Roseburia* in NewDMs. Comparison of ND (control) with KnownDM (case) network revealed *Dialister*, *Faecalibacterium*, *Haemophilus*, *Lachnospira*, *Phascolarctobacterium*, *Oscillospira*, and *Sutterella* as top driver nodes (high NESH score), followed by *Blautia*, *Akkermansia*, and *Streptococcus* with low NESH scores (Fig. 4C and Table S6). In KnownDMs, *Sutterella* was found to be associated with *Bacteroides*, *Bifidobacterium*, *Megasphaera*, and *Ruminococcus*, but in ND, it showed association only with *Bacteroides.* Similarly, the genus *Akkermansia* in KnownDMs was found to be associated with *Clostridium*, *Dialister*, and [*Eubacterium*], while in ND, it was found to be associated with many different genera along with *Clostridium* and [*Eubacterium*]. Thus, from these analyses, *Sutterella* was found to be a common driver genus across three disease groups. Besides driver genus analysis, identification of core hub communities among the four study groups was analyzed using NetShift workflow (detailed description of NetShift workflow used for this analysis is mentioned in Text S1 in the supplemental material) (Fig. S2A to C). We found significant change in core hub communities in NewDMs, while the core hub communities were similar in PreDMs and KnownDMs compared to ND.

10.1128/mSystems.00578-19.6 TABLE S6 Summary of NESH score and Jaccard score for driver nodes based on NetShift analysis. Download Table S6, PDF file, 0.2 MB. Copyright © 2020 Gaike et al. 2020 Gaike et al. This content is distributed under the terms of the Creative Commons Attribution 4.0 International license.

10.1128/mSystems.00578-19.9 FIG S2 Community shuffle plots representing core hub communities across four study groups based on positive microbial association networks. The plot is represented as a circle with an axis dividing it vertically into two parts. The left part represents the “control” and the right part represents the “case” subnetwork. Each node in both parts corresponds to their community affiliations in the respective networks. Node size in this plot corresponds to its coreness in the community. The edges connect similar nodes between the two halves (control and case), showing community shuffling. Download FIG S2, PDF file, 0.6 MB. Copyright © 2020 Gaike et al. 2020 Gaike et al. This content is distributed under the terms of the Creative Commons Attribution 4.0 International license.

10.1128/mSystems.00578-19.7 TEXT S1 Identification of core hub communities between four study groups based on microbial association networks. Download Text S1, PDF file, 0.09 MB. Copyright © 2020 Gaike et al. 2020 Gaike et al. This content is distributed under the terms of the Creative Commons Attribution 4.0 International license.

### Association of key taxa with biochemical parameters.

**FIG 5:**
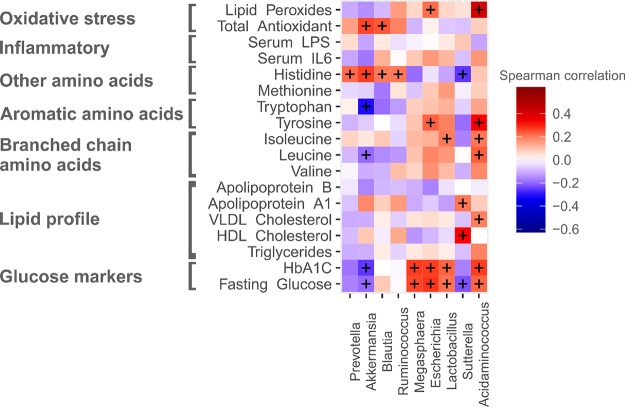
Spearman correlation analysis based on differentially abundant significant genera and significantly altered serum biomarkers. Spearman correlation values are shown in the vertical heatmap panel to the right. *P* values of <0.05 are indicated by the plus symbols.

For identifying the association of microbial taxa with significantly altered serum biomarkers (*P < *0.05), we selected nine significant (*P < *0.05) differentially abundant bacterial genera, namely, *Prevotella*, *Akkermansia*, *Blautia*, *Megasphaera*, *Escherichia*, *Lactobacillus*, *Ruminococcus*, *Sutterella*, and *Acidaminococcus*, that were found in all four groups and that were also identified as driver genera in NetShift analysis by using the Spearman correlation method (Fig. 5).

An abundance of *Prevotella* showed positive correlation with histidine (*P* = 0.025). *Prevotella* is one of the most dominant genera found in the Indian gut (19, 21, 22) and is inversely correlated with glucose, HbA1c, triglycerides, VLDL cholesterol, HDL cholesterol, leucine, tyrosine, methionine, IL-6, and lipid peroxides and positively correlated with total antioxidants; however, these associations were not found to be significant (*P > *0.05). An abundance of genus *Akkermansia* showed strong inverse correlation with fasting glucose (*P = *0.04), HbA1c (*P = *0.008), leucine (*P = *0.04), and tryptophan (*P = *0.002) and a strong positive correlation with histidine (*P = *0.006) and total antioxidants (*P = *0.007), whereas abundance of *Blautia* showed strong positive correlation with histidine (*P = *0.041) and total antioxidant (*P = *0.012). An abundance of *Ruminococcus* showed positive association with histidine (*P = *0.038), and an abundance of *Megasphaera* was positively associated with fasting glucose (*P = *0.007) and HbA1c (*P = *0.005). An abundance of the genus *Escherichia* was found to be positively correlated with fasting glucose (*P = *0.004), tyrosine (*P = *0.01), and lipid peroxides (*P = *0.026), while abundance of *Lactobacillus* showed positive correlation with fasting glucose (*P = *0.03), HbA1c (*P = *0.019), and isoleucine (*P = *0.04). An abundance of genus *Sutterella* was inversely associated with fasting glucose (*P = *0.02) and histidine (*P = *0.008) and positively associated with HDL cholesterol (*P = *0.002), while *Acidaminococcus* showed positive association with fasting glucose (*P = *0.02), VLDL cholesterol (*P = *0.04), leucine (*P = *0.01), isoleucine (*P = *0.03), tyrosine (*P = *0.0014), and lipid peroxides (*P = *0.00001) (Fig. 5).

## DISCUSSION

T2D is a widespread metabolic disorder that leads to various chronic health complications. Recently, the gut microbiome has been recognized as a major driver in the establishment of T2D. There are reports indicating a dysbiosis of gut microbiota in T2D subjects in Caucasian and Indian populations (4, 19). Vrieze et al. have reported that transfer of intestinal microbiota from lean donors to individuals with metabolic syndrome decreases insulin resistance (25).

Our earlier study has reported dysbiosis of gut microbiota in Indian diabetic subjects (19). In this study, we have analyzed the gut microbiome of PreDMs, NewDMs, KnownDMs on antidiabetic treatment, and ND individuals. Twenty-five different serum biomarkers were checked and compared with the gut microbiota to assess the different states of diabetes. Targeted 16S rRNA amplicon sequencing was used to assess the microbial diversity, community shuffling, and identification of driver taxa for the disease state. We have investigated relationships between a wide array of serum biomarkers responsible for progression of T2D with significantly diverged and differentially abundant taxa in each study group. Significantly different patterns were observed in the gut microbiota of PreDMs, NewDMs, and KnownDMs compared to ND. In KnownDMs, abundance of some microbial taxa was found to be similar to that of ND group.

Increased levels of BCAA and AA are found to be associated with insulin resistance, obesity, and T2D (26). Adams reported that BCAA and its metabolites are elevated in the blood of diabetic subjects (27), and their increased levels are associated with inflammation and insulin resistance, characteristics of T2D (10, 28). In our data, we found that BCAA and AAA remained elevated in both NewDMs and KnownDMs but not in PreDMs. We also found significantly low levels of histidine in NewDMs but not in PreDMs and KnownDMs. It has been reported that histidine supplementation in obese women with metabolic syndrome (29) and obese rats fed a high-fat diet (30) reduced insulin resistance, obesity, and metabolic syndrome by lowering inflammation and oxidative stress. Diabetic individuals are known to have a low-grade inflammation, and inflammatory markers are found to be elevated in their blood. We also found elevated levels of IL-6, an inflammatory cytokine (31), in NewDMs and KnownDMs but not in PreDMs, while LPS, a marker of low-grade inflammation, which induces metabolic endotoxemia (32) was found to be increased only in NewDMs. Since oxidative stress is known to be involved in the establishment of insulin resistance and diabetic complications (33), we measured total antioxidant capacity and lipid peroxides, a marker of oxidative damage to lipids in the blood. We found a significant decrease in total antioxidant capacity and increase in lipid peroxidation in treatment-naive NewDMs but not in PreDMs. In KnownDMs on treatment with metformin, an increase in total antioxidant capacity and decrease in lipid peroxidation were observed.

Earlier reports have demonstrated association of lower bacterial diversity with the disease condition (34, 35). In our study, a significantly lower number of observed OTUs was found in NewDMs compared to ND, which increased in KnownDMs on antidiabetic treatment (Fig. 1A). A lower alpha diversity in NewDMs and higher alpha diversity in KnownDMs suggests that there is loss of bacterial diversity in the disease condition, and interestingly, antidiabetic treatment helps in regaining bacterial diversity. Additionally, we have analyzed diversity of rare taxa to understand its community structure along with abundant taxa in different study groups. Interestingly, a higher number of rare taxa in KnownDMs were observed compared to ND and NewDMs. These results suggest that altered diversity of rare taxa may play an important role in structural as well as functional attributes of gut microbiota after antidiabetic treatment. On the basis of the results of beta diversity analysis, we found that the microbial diversity of prediabetics (PreDMs) is similar to that of nondiabetics (ND). However, the bacterial diversity of treatment-naive diabetics (NewDMs) was found to be different from that of nondiabetics (ND) and diabetics on antidiabetic treatment (KnownDMs). Interestingly, in KnownDMs, the microbial diversity is observed to be trending toward that of ND, probably due to antidiabetic treatment. Microbial diversity analysis at the phylum level revealed higher abundance of *Firmicutes* and *Proteobacteria* and decreased abundance of *Bacteroidetes* among NewDMs and KnownDMs, similar to earlier reports (19, 21).

At the genus level, microbial diversity analysis indicated that the levels of *Prevotella*, *Akkermansia*, *Megasphaera*, *Blautia*, *Lactobacillus*, *Escherichia*, *Ruminococcus*, *Sutterella*, and *Acidaminococcus* varied in the different study groups. Abundance of *Akkermansia* decreased significantly in NewDMs compared to ND. Decreased abundance of this mucin-degrading bacterial species is correlated with the onset of inflammation and metabolic disorders in mice (36, 37). Protein AMuc_1100 from *Akkermansia* or pasteurized bacterium has been linked to reduction in fat mass development, insulin resistance, and dyslipidemia in mice (38). Metformin treatment commonly prescribed for diabetes has also been linked with higher levels of *Akkermansia* in diabetic patients (39) due to enhancement of mucin-producing goblet cells (40). We did not find any change in the abundance of *Ruminococcus* in PreDMs, in contrast to the report of Ciubotaru et al. (41). Additionally, we observed decreased abundance of *Prevotella*, *Blautia*, and *Ruminococcus* and increased abundance of *Lactobacillus* in NewDMs. *Prevotella* is one of the dominant taxa in the Indian population (19, 21) and is known to be associated with a diet rich in plant-based polysaccharides (42, 43). *Prevotella* is also known to produce propionate, a short-chain fatty acid (SCFA) (44), which promotes reduction of hepatic lipogenesis and helps in the reduction of lipids in blood (45). Taken together, these observations may indicate that a high abundance of *Prevotella* in ND and low abundance in NewDMs can be a distinct biomarker of diabetes in the Indian population. In a recent study, it was reported that host genetics-driven changes in microbiome composition result in increased levels of SCFAs, such as propionate, which increases the risk of developing T2D, suggesting a causal relationship between microbiota and type 2 diabetes (46). This warrants conducting genetics-driven microbiome association studies in the Indian diabetic population to understand the functional impact of SCFAs on host metabolism at the population level. Among *Firmicutes*, we observed decreased abundance of *Blautia*, a known producer of short-chain fatty acids (47) in NewDMs. In KnownDMs, recovery of *Blautia* was probably associated with antidiabetic treatment, as described in a study on an Asian population (48). Observations of high abundance of *Lactobacillus* (3) and decreased abundance of *Akkermansia* in NewDMs corroborate previous findings (5). In KnownDMs, we found increased abundance of *Megasphaera*, *Escherichia*, and *Acidaminococcus* and decreased abundance of *Sutterella*, which is similar to earlier findings (6, 49–51). de la Cuesta-Zuluaga et al. reported that metformin treatment in diabetics is associated with increased abundance of *Megasphaera* in the Colombian population (39).

In gut microbiota, microbial community survives through their characteristics of mutualism and commensalism. During disease progression, the physiology of the host changes significantly, which affects the gut microbial community and their interaction pattern. Under these circumstances, some microbes act as key players in the community, known as driver microbes (24). Different microbes interacting with each other in the community constitute core taxa. We analyzed positive associations among highly abundant genera in each group. NetShift analysis of core hub communities revealed that ND subjects have the maximum number of core hubs representing common genera, which changed significantly in NewDMs. In PreDMs, in addition to core hubs observed in ND, *Sutterella* was identified as an additional core hub. Earlier reports have suggested that the genus *Sutterella* is found to be associated with many diseases such as type 1 diabetes and inflammatory bowel disease (IBD) (50, 51). In KnownDMs, core hub communities were found to be similar to ND. Increased abundance of genus *Sutterella* has been reported earlier in prediabetic gut microbiota (52). We found that *Sutterella* was a major and common driver across all disease groups.

Further, we analyzed correlation of microbiota with biochemical parameters measured to assess the status of diabetes. We observed significant decrease in total antioxidant capacity and increase in lipid peroxides in NewDMs compared to ND. The abundance of *Akkermansia* was positively correlated with total antioxidant capacity and inversely correlated with lipid peroxides in all groups. Administration of live or attenuated *Akkermansia* to diabetic rats led to decrease in oxidative stress, lipotoxicity, GLP-1, LPS, inflammation, and increase in HDL and improvement in liver function (53). We did not find any significant inverse association of *Akkermansia* and inflammatory markers, although *Akkermansia* is reported to reduce low-grade inflammation (36). We observed a strong inverse association of *Akkermansia* with glucose and HbA1c, similar to those reported by Schneeberger et al. (36). Recently, administration of *Akkermansia* has been shown to improve glucose homeostasis in mice fed a HFD (high-fat diet) (40). A significant association between the genus *Prevotella*, the most abundant genus in the Indian gut, and parameters such as glucose, lipids, BCAA, and AAA was not observed. A study by Pedersen et al. (17) demonstrated a positive association of *Prevotella* with BCAA and suggested that increased levels of circulating BCCA are due to the high prevalence of Prevotella copri, which was not found in our study. We observed a higher level of *Prevotella* in ND than in NewDMs. Kovatcheva-Datchary et al. (54) demonstrated that consumption of a diet rich in plant-derived fibers improved glucose metabolism through increased abundance of *Prevotella* in the Caucasian responder group and that increasing *Prevotella* by fecal transplantation improved glucose metabolism in germfree mice. Previously, an increased abundance of *Lactobacillus* in Indian type 2 diabetic patients (19) and a positive correlation between *Lactobacillus*-derived metagenomic clusters with fasting glucose and HbA1c was observed in Caucasian type 2 diabetic patients (4). In our study, we also find a positive correlation between *Lactobacillus* abundance with glucose and HbA1c level. This could be due to the higher genetic potential of *Lactobacillus* to utilize carbohydrates (55). However, analysis at lower taxonomic level such as species or strain is required, since probiotic strains of *Lactobacillus* are reported to be beneficial for lowering blood glucose (56). In our study, we found increased abundance of *Escherichia* in KnownDMs, which was positively correlated with blood metabolites such as glucose, tyrosine, and lipid peroxides. It is known that metformin, which is commonly used as an antidiabetic agent, leads to disturbance of the intestinal microbiota and increases in the abundance of opportunistic pathogens such as *Escherichia* (6, 57). The increased abundance of genus *Escherichia* observed in our KnownDMs was possibly due to metformin. Further investigations are necessary to understand its positive correlation with blood metabolites in diabetic subjects.

Thus, this study gives us insights into the altered microbial community composition among different diabetic groups compared to ND and their association with clinical biomarkers in the Indian population. We are aware that the key limitation of this study is the sample size for PreDMs and NewDMs compared to both ND and KnownDMs. A larger study with more samples would help to generalize these findings. We also propose comparing prospectively gut microbiota changes in the same patient group before and after therapeutic introduction and to match it with prediabetic and nondiabetic subjects in future studies.

### Conclusions.

Our findings show differences in the gut microbiome in PreDMs, NewDMs, and KnownDMs compared to ND. In PreDMs, the gut microbiome does not change significantly from that of ND, whereas in NewDMs, both the abundance and diversity changed significantly, which in KnownDMs on antidiabetic treatment seems to be restored to some extent.

## MATERIALS AND METHODS

### Study population and sample collection.

This is a retrospective study using a total of 102 subjects from the western region of India who were selected for this study during 2015 to 2016. All subjects were 30 to 60 years old. Healthy subjects with HbA1c of ≤5.7% were termed nondiabetic subjects (ND) (*n* = 35). Diabetic subjects with antidiabetic treatment for at least the past year with HbA1c of ≥6.5% were termed known diabetes mellitus subjects (KnownDMs) (*n* = 39). Newly diagnosed diabetic subjects who were not on any antidiabetic medication with HbA1c of ≥6.5% were termed newly diagnosed diabetes mellitus subjects (New-DMs) (*n* = 11, of which *n* = 5 are obese), and prediabetic subjects with HbA1c of 5.7% to 6.4% were termed prediabetics (PreDMs) (*n* = 17). All the study groups were differentiated based on the HbA1c level by ADA (American Diabetes Association) guidelines (58). The study and the experimental protocols were approved by the institutional ethical committee of the National Centre for Cell Science (NCCS) (Pune, India), and informed consent and metadata were obtained from all participants.

The exclusion criteria for all four groups included antibiotic consumption in the last 3 months, any major gastrointestinal surgery, and presence of any known chronic or clinical disorder. All participants were screened before sampling, and an early morning stool sample was collected on the following day in a sterile stool container. Early morning fasting blood sample was also collected on the same day by phlebotomists from the clinical laboratory (Golwilkar Metropolis, Pune, India) to assess serum biomarkers. Fecal samples from all the subjects were collected and stored at −80°C until further processing, whereas blood samples were processed immediately.

### Biochemical analysis.

Fasting plasma glucose and glycated hemoglobin (HbA1c) were measured using hexokinase and high-performance liquid chromatography (HPLC) (Tosoh Bioscience, USA) method, respectively. Total cholesterol, triglycerides, and HDL cholesterol were measured by the serum enzymatic method. Apolipoproteins A1 and B were estimated by serum nephelometry (BN ProsPec system, Siemens, Germany). Vitamin B_12_, folic acid, and homocysteine were measured using competitive-binding immunoenzymatic assay. All measurements were done on an autoanalyzer (Architect Integrated CI- 2800; Abbott, USA) at Golwilkar Metropolis, Pune, India. IL-6 and LPS levels in serum were estimated using a human IL-6 Quantikine high-sensitivity (HS) enzyme-linked immunosorbent assay (ELISA) kit (catalog no. HS600B; R&D Systems, MN, USA) and LPS ELISA kit (catalog no. CEB52Ge; Cloud Clone Corp, USA). Serum samples diluted 1:100 were used to measure adiponectin by ELISA (catalog no. DRP 300; R&D Systems, MN, USA). Blood plasma samples were deproteinated using sulfosalicylic acid (SSA). Deproteinated samples were used for the quantification of plasma amino acids by HPLC coupled with solvent delivery systems, autosampler, and photodiode array detector (all from Agilent 1100 series, Agilent Technology, Germany). A precolumn derivatization was done for analysis of the amino acids using a derivatizing agent, *o*-phthalaldehyde. From serum samples, assessment of total antioxidants was performed and measured spectrophotometrically at 450 nm by the protocol of Kambayashi et al. (59). Lipid peroxides were measured in plasma in nanomoles of malondialdehydes formed by the protocol of Acharya et al. (60).

### DNA extraction and 16S rRNA gene amplicon sequencing.

Total community DNA was extracted from all 102 samples using QIAamp stool DNA minikit (Qiagen, Germany) per the manufacturer’s instructions. DNA was quantified using NanoDrop (ND-1000; Thermo Fisher Scientific, USA), and the quality of DNA was checked by gel electrophoresis. The DNA samples were subjected to amplification of 16S rRNA gene using V4 region-specific primers (V4 Forward [5′GTGCCAGCMGCCGCGGTAA3′] and V4 Reverse [5′GGACTACHVGGGTWTCTAAT3′]) (61). PCR was performed using the following conditions: initial denaturation at 95°C for 3 min; 25 cycles with 1 cycle consisting of 95°C for 30 s, 55°C for 30 s, and 72°C for 30 s; and a final extension step at 72°C for 7 min (61). A 2.5-μl DNA (5-ng/μl concentration) sample was used as a template in each 25-μl PCR mixture. After amplification, products were cleaned using AMPure XP beads (catalog no. A63882; Beckman Coulter, Inc., USA) and subjected to library preparation using NextraXT library preparation kit (Illumina, USA) followed by limited cycle PCR to enrich the adapter ligated DNA molecules. Final cleanup was performed using AMPure XP beads to obtain libraries which were assessed for fragment size distribution using TapeStation (catalog no. 5067-5582; Agilent Technologies, USA) and were quantified using Qubit DNA (catalog no. Q32854; Thermo Fisher Scientific, USA) before sequencing. The quantified libraries were clonally amplified on cBOT and sequenced using Illumina HiSeq 2500 (Illumina Inc., USA) with 2 × 250 bp paired-end chemistry. Sequences retrieved from Illumina HiSeq sequencing are available at the NIH Sequence Read Archive (SRA) under the Bioproject identifier (ID) or accession no. PRJNA448494.

### Bioinformatic analysis.

Paired-end reads were assembled using PEAR v0.9.10 software (62). The assembled reads were trimmed by using cutadapt version 1.13 (63) to remove adapter sequences from both ends. The quality filtered sequences were used for further analysis using Quantitative Insights Into Microbial Ecology (QIIME) v. 1.9 (64). Operational taxonomic units (OTUs) were binned by using closed reference OTU picking strategy using UCLUST algorithm (64) at 97% sequence similarity using Greengenes database v13_8 (65). Representative sequences from each OTU were used for taxonomic assignment using the RDP classifier (66). Singletons were removed from the OTU table, and the OTU table was normalized for the least number of sequences (86,770 sequences per sample) and used for downstream analysis. To calculate the *Firmicutes*-to-*Bacteroidetes* ratio, the formula used was $\text{ratio}=\left(\frac{A}{B}:\frac{B}{B}\right)$ where *A* is mean abundance for *Bacteroidetes* and *B* is mean abundance for *Firmicutes*. The ratio for each sample was calculated, and the average ratio is mentioned for each group.

### Random forest analysis.

Genera and metabolites important for differentiating disease status were identified using random forest algorithm. The top 30 most abundant genera present in all samples and serum metabolites were included for analysis. The ranking of genera and serum metabolites according to mean decrease in accuracy (mean decrease Gini score) were obtained from the random forest algorithm using default parameters in the R 3.4.0. environment “randomForest” (with ntree = 1,000), as mentioned in previous reports (67, 68).

### NetShift analysis.

CCREPE (version 1.7.0) analysis (http://huttenhower.sph.harvard.edu/ccrepe) was performed separately for all study groups to identify the significant (*P* < 0.05) positive correlations among the highly abundant genera, which resulted in a positive edgelist. This edgelist is further used to analyze the driver microbes and core hub genera of study groups using the NetShift tool (24) available at https://web.rniapps.net/netshift/index_file.php.

### Statistical analysis.

All biochemical parameters were analyzed using a nonparametric Mann-Whitney test to understand the comparison between two study groups, which were compared one at a time. The differentially abundant genera were analyzed by mean difference with the false-discovery rate (FDR) correction using the discrete FDR (DS-FDR) method (69). Kruskal-Wallis test followed by FDR correction was applied to OTU table to derive differentially abundant diabetes-related biomarkers (OTUs) using the QIIME command *group_significance.py*. A generalized UniFrac distance was performed using GUniFrac Package (20) to identify the differences among four study groups, i.e., ND, PreDMs, NewDMs, and KnownDMs, and statistical test permutational multivariate analysis of variance (PERMANOVA) was performed using the vegan package in R (https://cran.r-project.org or https://github.com/vegandevs/vegan). Spearman correlation was performed to identify associations among biochemical parameters and microbial genera using R package Hmisc (https://cran.r-project.org/web/packages/Hmisc/index.html), and visualization was done using ggplot2 package in R (70).

### Data availability.

The data sets generated and/or analyzed during the current study are available in the following repositories. Raw data are available on NIH Sequence Read Archive (SRA) under the Bioproject ID PRJNA448494. To enable future analysis, metadata and OTU table data are available at https://github.com/aksbiome/Type-2-Diabetes-and-gut-microbiome.
