# Supplementary material for: The Gut Microbial Diversity of Newly Diagnosed Diabetics but Not of Prediabetics Is Significantly Different from That of Healthy Nondiabetics
Source: mSystems. 2020 Mar 31;5(2):e00578-19. doi: 10.1128/mSystems.00578-19 (PMC7112960; doi:10.1128/mSystems.00578-19)
Supplement: TABLE S1 [file mSystems.00578-19-st001.pdf]

Table S1: Sequence statistics for bioinformatics analysis

Sample ID	Raw Seqs	Assembled Seq	Filter_fastq_fna	nonChimeraCalls	Biom_Summary_Seqs
UNI001	325808	317774	262825	207937	148052
UNI002	483708	481520	438380	403352	351695
UNI003	502109	479608	336241	289227	217603
UNI004	432196	408085	264841	205391	139160
UNI005	501300	492854	434740	397243	308504
UNI006	262643	247356	145762	130224	110919
UNI007	397721	381351	243483	187878	130530
UNI008	268229	257278	192409	161010	124209
UNI009	341676	319247	182539	138643	86778
UNI010	373345	355098	253784	204204	161663
UNI011	942157	914502	752616	628196	476638
UNI012	312106	300483	250564	214338	178028
UNI013	607469	600084	550815	487278	427145
UNI014	612594	602440	514061	442649	363787
UNI015	614672	601640	512106	417922	292855
UNI016	580143	567036	475765	397370	267412
UNI017	562190	553668	473992	399444	335125
UNI018	613178	601606	536431	443440	347472
UNI019	674976	661651	589054	480371	376404
UNI020	318451	303823	246197	210245	146343
UNI021	599093	593670	551006	455459	346339
UNI022	476066	470585	433569	399641	354007
UNI023	656590	648535	603282	443471	307675
UNI024	592796	583006	526267	470225	375338
UNI025	938539	910019	667171	589981	456698
UNI026	258950	253392	230337	182196	131758
UNI027	296093	290551	258157	196214	130998
UNI028	467761	460007	404441	340552	214539
UNI029	746467	729820	589339	448514	225929
UNI030	351316	345642	295603	212577	154796
UNI031	254741	247618	222868	164376	98920
UNI032	1060066	901000	703428	618172	471745
UNI033	465996	458880	396801	321288	174942
UNI034	1129829	986552	758044	651649	492148
UNI035	428054	423484	369052	274069	172575
UNI036	556940	549895	477830	328985	189160
UNI037	583820	576211	498555	413840	268241
UNI038	650866	627499	495956	448805	368407
UNI039	529730	523831	456639	316176	167760
UNI040	558996	548698	451897	307237	196876
UNI041	426750	419207	356630	279454	191111
UNI042	505673	418812	334122	249791	157539
UNI043	479656	474551	409604	319438	222066
UNI044	438923	433894	374677	242635	143227

UNI045	485329	477847	409797	332510	219605
UNI046	419753	412981	354307	298875	208622
UNI047	408957	405276	355212	240433	149934
UNI048	463773	455918	386034	271508	194482
UNI049	466237	455500	378861	309488	212528
UNI050	298474	287820	253768	188862	133477
UNI051	619555	608349	505688	300645	174814
UNI052	978780	888733	711764	639491	504859
UNI053	1557515	1422603	1123214	1025484	834317
UNI055	524980	515957	441767	343757	244899
UNI056	465171	446692	354476	241940	176744
UNI057	430560	424257	358883	278009	157796
UNI058	489911	477182	397854	291429	206057
UNI059	555051	537879	441433	315840	169119
UNI061	300172	287169	251575	183138	127021
UNI062	413398	405471	334803	238320	156469
UNI063	325354	320320	266225	185705	132161
UNI064	552490	542777	451155	303819	210331
UNI066	352202	348157	294810	207603	132367
UNI067	461843	452229	370774	257355	183960
UNI068	405111	392411	303577	244645	147989
UNI069	334189	322093	271780	208761	140503
UNI071	1053289	902332	733227	706867	650284
UNI072	481236	473068	376956	301160	154425
UNI074	584889	570031	448883	350312	237095
UNI075	445242	436726	350009	244757	183253
UNI076	533537	518408	444407	324653	220062
UNI077	467347	450009	345392	258624	178777
UNI078	669923	666798	590502	408400	328186
UNI080	1065370	923652	725331	622733	576486
UNI081	507814	499053	440009	305845	208940
UNI082	594903	576922	466167	328998	198034
UNI083	455860	427943	374781	303121	258844
UNI084	378129	373653	333504	226998	126040
UNI085	325372	320820	280681	210236	133330
UNI087	697285	657180	537975	461305	348749
UNI090	720269	669639	563985	472708	398147
UNI091	1191033	1099589	899421	775722	603310
UNI092	995037	936620	809177	741980	658507
UNI093	1063258	898635	736022	686593	625858
UNI094	944921	835281	678606	583604	443912
UNI095	1085820	991546	826107	727521	557935
UNI096	673924	641175	542430	491470	443372
UNI097	1489577	1390519	1150192	958341	734747
UNI098	493141	473496	398585	353577	292826
UNI101	1288744	1246341	1127401	996590	677571
UNI102	1320069	1274565	1153626	985735	763401

UNI103	1061633	1024561	925979	758727	606719
UNI104	778363	752264	676345	564162	406354
UNI105	1008650	975857	882415	790085	579880
UNI106	1062156	1029001	931629	859384	670110
UNI107	1205907	1170208	1050754	875982	667837
UNI108	1351104	1305182	1175807	902221	534575
UNI109	996742	963692	873780	781028	665789
UNI110	1050025	1015930	913223	790328	593221
UNI111	799313	772191	698712	606041	500007
UNI112	1548277	1495505	1339954	1208573	871401
UNI113	1112830	1070558	969214	909785	734429
