# Supplementary figures and images for: The Gut Microbial Diversity of Newly Diagnosed Diabetics but Not of Prediabetics Is Significantly Different from That of Healthy Nondiabetics
Source: mSystems. 2020 Mar 31;5(2):e00578-19. doi: 10.1128/mSystems.00578-19 (PMC7112960; doi:10.1128/mSystems.00578-19)

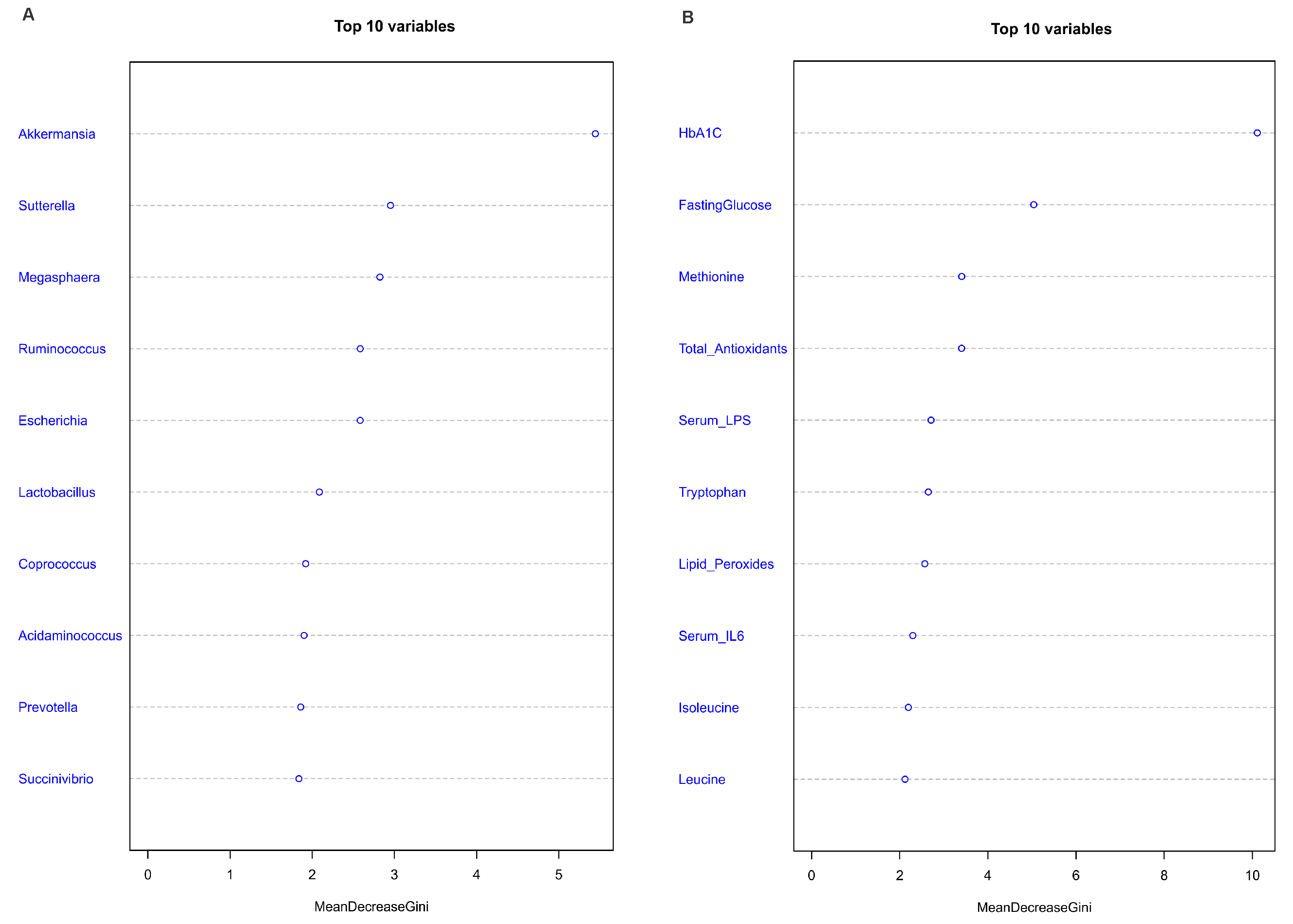

Supplement: FIG S1 [file mSystems.00578-19-sf001.tif]

**Fig. S2 A**

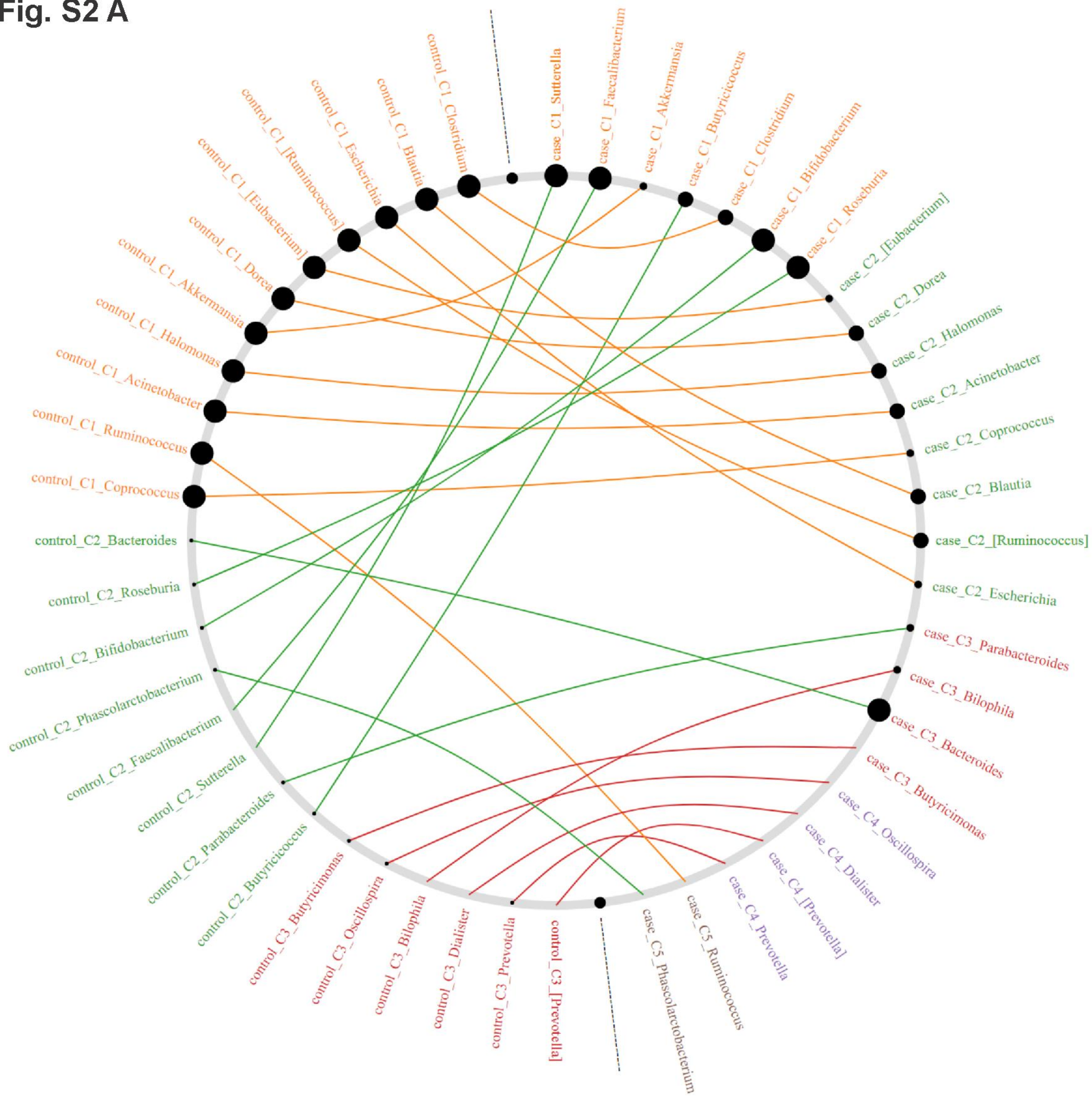

Fig. S2 B

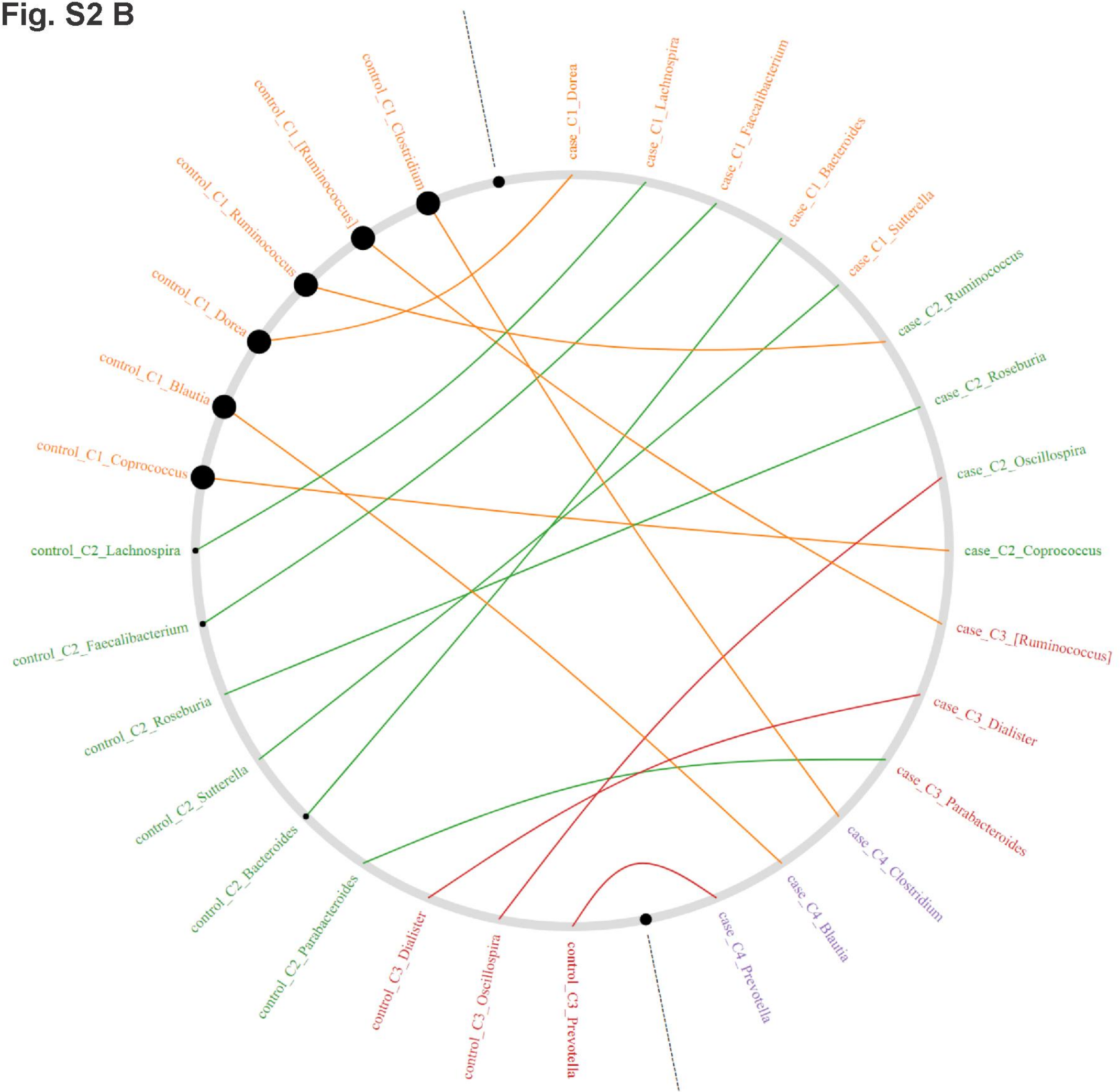

Fig. S2 C

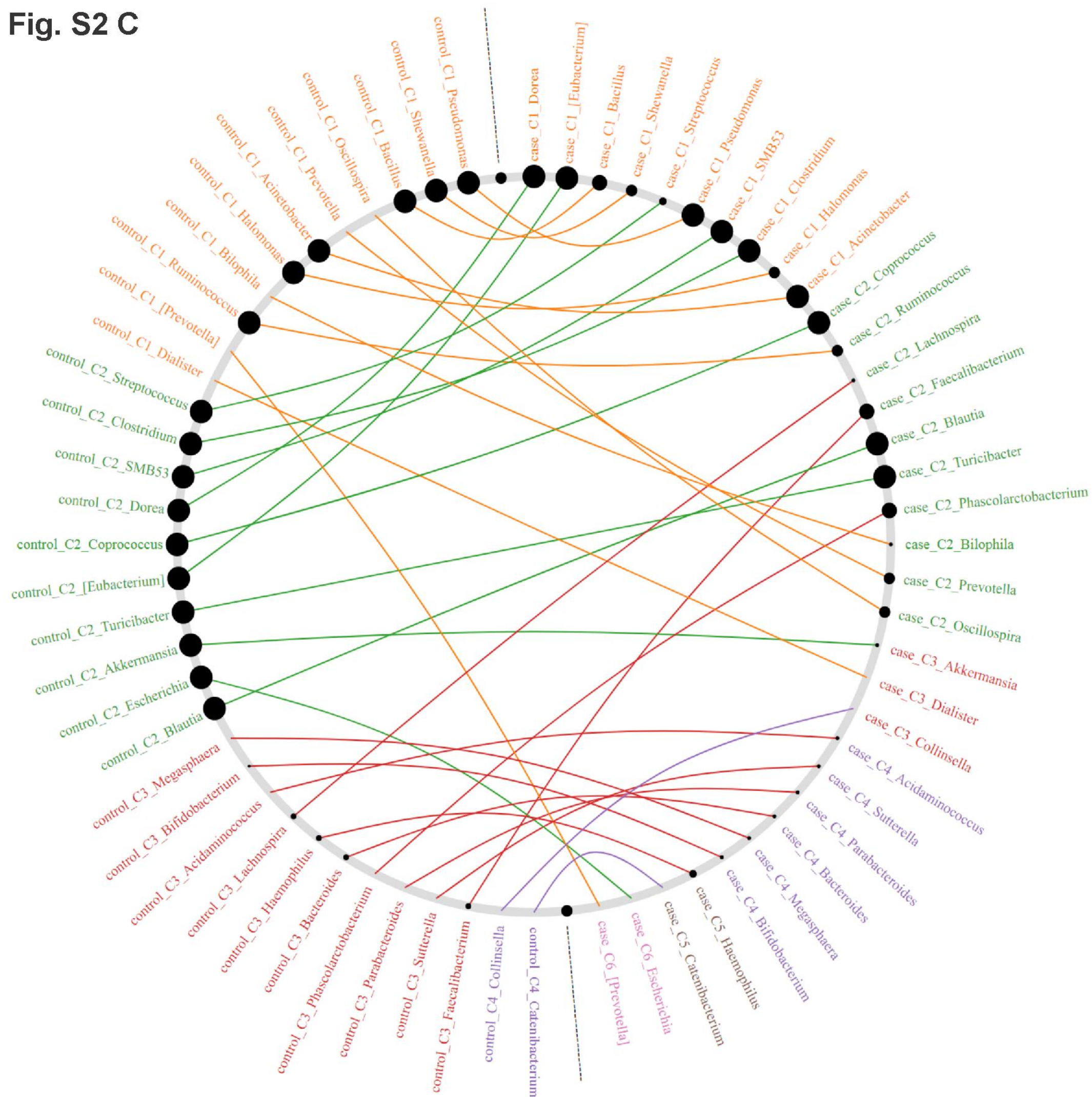

Supplement: FIG S2 [file mSystems.00578-19-sf002.pdf]
