# Supplementary material for: The Gut Microbial Diversity of Newly Diagnosed Diabetics but Not of Prediabetics Is Significantly Different from That of Healthy Nondiabetics
Source: mSystems. 2020 Mar 31;5(2):e00578-19. doi: 10.1128/mSystems.00578-19 (PMC7112960; doi:10.1128/mSystems.00578-19)
Supplement: TABLE S5 [file mSystems.00578-19-st005.pdf]

Table S5 : Distribution of rare OTUs across four study groups

Sr. No.	Taxonomy	ND Rare OTUs %
1	Bacteroidetes	64.09225114
3	Firmicutes	17.65432127
7	Proteobacteria	6.040022573
2	Verrucomicrobia	5.989933739
4	Actinobacteria	2.897845018
6	Cyanobacteria	1.9811662
5	Elusimicrobia	1.073389111
8	Lentisphaerae	0.117449681
9	Synergistetes	0.04852771
11	Tenericutes	0.036071933
10	Spirochaetes	0.02075963
12	Crenarchaeota	0.012123624
13	[Thermi]	0.009798545
17	Acidobacteria	0.006676297
18	Nitrospirae	0.005148388
20	Chloroflexi	0.004982311
14	Planctomycetes	0.004085495
15	Fusobacteria	0.001727201
16	NC10	0.001727201
19	TM6	0.001195755
21	Gemmatimonadetes	0.00079717

Sr. No.	Taxonomy	PreDMs Rare OTUs %
1	Bacteroidetes	61.99431899
2	Firmicutes	22.26860734
3	Proteobacteria	8.182144818
4	Actinobacteria	3.404605821
5	Cyanobacteria	2.001640578
6	Verrucomicrobia	1.133151198
7	Elusimicrobia	0.661044411
8	Lentisphaerae	0.166159353
9	Tenericutes	0.08962165
10	[Thermi]	0.036743521
11	Synergistetes	0.026439065
12	Chloroflexi	0.008541852
13	Fusobacteria	0.007728342
14	Acidobacteria	0.004948851
15	FBP	0.003457416
16	Spirochaetes	0.002508322
17	Planctomycetes	0.002101567
18	TM7	0.001559227
19	Nitrospirae	0.001423642
20	Euryarchaeota	0.00108468
21	Crenarchaeota	0.001016887
22	GAL15	0.000338962
23	Gemmatimonadet	6.77925E-05

Sr. No.	Taxonomy	NewDMs Rare OTUs %
1	Bacteroidetes	41.31654216
3	Firmicutes	38.46668832
2	Proteobacteria	16.14309512
5	Actinobacteria	2.659067336
4	Cyanobacteria	0.906995505
6	Tenericutes	0.289375255
7	Elusimicrobia	0.194558237
8	Euryarchaeota	0.013620124
9	Lentisphaerae	0.006705292
10	Fusobacteria	0.001571553
11	TM7	0.001047702
13	Verrucomicrobia	0.000314311
12	Synergistetes	0.000314311
14	[Thermi]	0.00010477

Sr. No.	Taxonomy	KnownDMs Rare OTUs %
1	Firmicutes	55.1049318
2	Proteobacteria	20.06765739
3	Bacteroidetes	13.87723439
4	Actinobacteria	6.838666859
5	Cyanobacteria	0.803807087
6	Verrucomicrobia	0.483889573
7	Acidobacteria	0.424645589
8	Crenarchaeota	0.423881151
9	Planctomycetes	0.377632621
10	[Thermi]	0.327561899
11	Tenericutes	0.318770856
12	Nitrospirae	0.24003369
13	Chloroflexi	0.215953877
14	Lentisphaerae	0.091350401
15	Euryarchaeota	0.089821524
16	Gemmatimonadet	0.060008423
17	Synergistetes	0.055421792
18	Fusobacteria	0.043572995

19	TM7	0.042044118
20	NC10	0.028284225
21	WS3	0.017582086
22	WPS-2	0.012231016
23	GAL15	0.011848797
24	KSB3	0.009555481
25	Thermotogae	0.009555481
26	Spirochaetes	0.00535107
27	SBR1093	0.004586631
28	Deferribacteres	0.004586631
29	Elusimicrobia	0.003822193
30	Chlorobi	0.002293316
31	Armatimonadetes	0.001528877
32	GN02	0.000764439
33	AD3	0.000764439
34	NKB19	0.000382219
