# Supplementary material for: The Gut Microbial Diversity of Newly Diagnosed Diabetics but Not of Prediabetics Is Significantly Different from That of Healthy Nondiabetics
Source: mSystems. 2020 Mar 31;5(2):e00578-19. doi: 10.1128/mSystems.00578-19 (PMC7112960; doi:10.1128/mSystems.00578-19)
Supplement: TABLE S6 [file mSystems.00578-19-st006.pdf]

Table S6: Summary of NESH score and Jaccard score for Driver nodes based on NetShift Analysis

ND vs PreDMs	S_ID	Jaccard-score	NESH-score
	[Eubacterium]	0.25	0.75
	[Prevotella]	1	0
	[Ruminococcus]	0.545	0.886
	Acinetobacter	0.222	1.014
	Akkermansia	0	1.472
	Bacteroides	0.625	1.125
	Bifidobacterium	0.333	1.833
	Bilophila	0	1.917
	Blautia	0.308	1.298
	Butyricicoccus	0	2.511
	Butyricimonas	0	1.458
	Clostridium	0	1.606
	Coprococcus	0.3	0.7
	Dialister	1	0
	Dorea	0.333	1.083
	Escherichia	0.375	0.625
	Faecalibacterium	0.125	2.625
	Halomonas	0.444	0.556
	Oscillospira	0.5	0.5
	Parabacteroides	0.25	1.5
	Phascolarctobacterium	0	1.75
	Prevotella	0.6	0.4
	Roseburia	0.4	1.25
	Ruminococcus	0	1.225
	Sutterella	0.2	2.1

ND vs NewDMs	S_ID	Jaccard-score	NESH-score
	[Ruminococcus]	0	1.667
	Bacteroides	0.4	0.6
	Blautia	0	1.667
	Clostridium	0	1.667
	Coprococcus	0	1.667
	Dialister	0	2
	Dorea	0	1.667
	Faecalibacterium	0.5	0.5
	Lachnospira	0.333	1.5
	Oscillospira	0	2
	Parabacteroides	0	2.667
	Prevotella	0	2.5
	Roseburia	0	2.667
	Ruminococcus	0	2.286

Sutterella	0	2.667

ND vs KnownDMs	S_ID	Jaccard-score	NESH-score
	[Eubacterium]	0.538	0.942
	[Prevotella]	0.5	1.083
	Acidaminococcus	0	1.833
	Acinetobacter	0.615	0.865
	Akkermansia	0.2	0.983
	Bacillus	0.417	0.917
	Bacteroides	0.375	0.833
	Bifidobacterium	0.2	1.65
	Bilophila	0.333	1.083
	Blautia	0.368	1.175
	Catenibacterium	0	1.583
	Clostridium	0.688	0.458
	Collinsella	0	1.583
	Coprococcus	0.421	1.123
	Dialister	0	1.833
	Dorea	0.529	0.755
	Escherichia	0	1.155
	Faecalibacterium	0.111	2.056
	Haemophilus	0.143	1.536
	Halomonas	0.4	0.6
	Lachnospira	0	1.567
	Megasphaera	0	1.667
	Oscillospira	0.25	1.75
	Parabacteroides	0.25	1.417
	Phascolarctobacterium	0	2.65
	Prevotella	0.375	1.458
	Pseudomonas	0.467	1.133
	Ruminococcus	0.214	1.095
	Shewanella	0.4	0.6
	SMB53	0.462	1.179
	Streptococcus	0.182	1.167
	Sutterella	0.25	1.75
	Turicibacter	0.467	1.283
