# Supplementary material for: The Gut Microbial Diversity of Newly Diagnosed Diabetics but Not of Prediabetics Is Significantly Different from That of Healthy Nondiabetics
Source: mSystems. 2020 Mar 31;5(2):e00578-19. doi: 10.1128/mSystems.00578-19 (PMC7112960; doi:10.1128/mSystems.00578-19)
Supplement: TEXT S1 [file mSystems.00578-19-s0001.pdf]

Text S1

Identification of core hub communities between four study groups based on microbial association networks

We generated microbial association networks for ND, PreDMs, NewDMs, and KnownDMs followed by mining only statistically significant (p<0.05) positive association networks separately using CCREPE (Compositionality Corrected by REnormalization and PErmutation) tool (http://huttenhower.sph.harvard.edu/ccrepe). We then applied NetShift workflow using the specific webserver (https://web.rniapps.net/netshift/) [21] and generated comparative multiple association network between the groups where ND is the control network and remaining are the case networks. The NetShift workflow compares only common networks from two groups for further analysis [21]. In this comparison we observed low jaccard edge index (0.258) representing satisfactory rewiring in the shuffling plots between ND (Control) and PreDMs (Case). This network revealed two different core hub communities. ND (Control) core hub has genera such as *Acinetobacter, Akkermansia, Blautia, Clostridium, Coprococcus, Dorea, Escherichia, Halomonas, Ruminococcus, [Ruminococcus]* and *[Eubacterium]* which were shifted to *Bacteroides, Bifidobacterium, Faecalibacterium, Roseburia* and *Sutterella* as the core hub nodes in case (PreDMs) network [Fig. S2 A]. Very low jaccard edge index (0.062) was observed between ND (Control) and NewDMs (Case). This network revealed core hub community only in ND (Control) group as, *Blautia, Coprococcus, Clostridium, Dorea, Ruminococcus,* and *[Ruminococcus]* [Fig. S2 B]. Further, we compared ND (Control) network with KnownDMs (Case) network and observed high jaccard edge index (0.342) which suggests low rewiring between shuffling plots. This network revealed two core hub communities consisting of genera such as *Acinetobacter, Akkermansia, Bacillus, Blautia, Clostridium, Coprococcus, Dorea,*

Escherichia, Halomonas, Pseudomonas, Ruminococcus, Shewanella, SMB53, Streptococcus, Turibacter and *[Eubacterium]* in ND. In KnownDMs (case) network core hub genera included *Acinetobacter, Blautia, Clostridium, Coprococcus, Dorea*, *Pseudomonas, SMB53, Turibacter* and *[Eubacterium]*. In KnownDMs network core hub genera was similar to that of ND except *Akkermansia*, *Escherichia, Halomonas, Ruminococcus* and *Streptococcus* [Fig. S2 C]. Thus these comparative network analysis revealed that *Blautia*, *Clostridium*, *Coprococcus*, *Dorea* and *Ruminococcus* which are known commensals contributing to maintenance of gut homeostasis, are common core hub communities representing positive association networks.
